# Implementation of internet-delivered cognitive behavior therapy within community mental health clinics: a process evaluation using the consolidated framework for implementation research

**DOI:** 10.1186/s12888-017-1496-7

**Published:** 2017-09-12

**Authors:** H.D. Hadjistavropoulos, M.M. Nugent, D. Dirkse, N. Pugh

**Affiliations:** Department of Psychology, University of Regina, 3737 Wascana Parkway, Regina, SK S4S 0A2 Canada

**Keywords:** Process evaluation, Implementation research, Consolidated framework for implementation research, Internet-delivered cognitive behavior therapy, Depression, Anxiety

## Abstract

**Background:**

Depression and anxiety are prevalent and under treated conditions that create enormous burden for the patient and the health system. Internet-delivered cognitive behavior therapy (ICBT) improves patient access to treatment by providing therapeutic information via the Internet, presented in sequential lessons, accompanied by brief weekly therapist support. While there is growing research supporting ICBT, use of ICBT within community mental health clinics is limited. In a recent trial, an external unit specializing in ICBT facilitated use of ICBT in community mental health clinics in one Canadian province (ISRCTN42729166; registered November 5, 2013). Patient outcomes were very promising and uptake was encouraging. This paper reports on a parallel process evaluation designed to understand facilitators and barriers impacting the uptake and implementation of ICBT.

**Methods:**

Therapists (*n* = 22) and managers (*n* = 11) from seven community mental health clinics dispersed across one Canadian province who were involved in implementing ICBT over ~2 years completed an online survey (including open and closed-ended questions) about ICBT experiences. The questions were based on the Consolidated Framework for Implementation Research (CFIR), which outlines diverse constructs that have the potential to impact program implementation.

**Results:**

Analyses suggested ICBT implementation was perceived to be most prominently facilitated by intervention characteristics (namely the relative advantages of ICBT compared to face-to-face therapy, the quality of the ICBT program that was delivered, and evidence supporting ICBT) and implementation processes (namely the use of an external facilitation unit that aided with engaging patients, therapists, and managers and ICBT implementation). The inner setting was identified as the most significant barrier to implementation as a result of limited resources for ICBT combined with greater priority given to face-to-face care.

**Conclusions:**

The results contribute to understanding facilitators and barriers to using ICBT within community mental health clinics and serve to identify recommendations for improving uptake and implementation of ICBT in clinic settings.

**Electronic supplementary material:**

The online version of this article (10.1186/s12888-017-1496-7) contains supplementary material, which is available to authorized users.

## Background

Despite being highly prevalent and disabling conditions [1], depression and anxiety often remain untreated [2]. Barriers to treatment are diverse and include limited perceived need for treatment, preference to independently manage problems, inadequate finances, time constraints, transportation or mobility challenges, poor access to providers, or concerns about privacy and stigma [2]. Internet-delivered cognitive behavior therapy (ICBT) for depression and anxiety has the potential to address many of these barriers and improve patient access to evidence-based treatment [3]. ICBT involves patients reviewing structured weekly lessons delivered via the Internet over the course of several months. Homework is assigned to facilitate learning and brief therapist support is typically provided using emails or telephone calls. Randomized controlled trials provide strong support for ICBT with large treatment effects identified for those receiving ICBT [4]; these promising findings extend to when ICBT is delivered in routine practice settings [5]. Nevertheless, this latter research primarily has been conducted in specialized ICBT clinics, where there are limited competing demands on therapists’ time [5].

At present, ICBT is largely unavailable on a routine basis to most Canadian patients who have depression and anxiety [3]. In 2014, the Mental Health Commission of Canada released a briefing document encouraging broader use of technology in mental health care [6]. Consistent with this call, a recent trial by our group compared ICBT for depression and anxiety when delivered by therapists working in a specialized clinic to when ICBT was delivered by therapists working in community mental health clinics distributed across one Canadian province (i.e., clinics where therapists primarily deliver face-to-face care but secondarily use ICBT) [7]. Supporting the public health potential of ICBT, regardless of setting, completion rates, satisfaction, and outcomes were very strong. In both the specialized clinic and community mental health clinics, 78% of patients completed all core ICBT lessons, over 94% reported that the course was worth their time, and large effects were found from pre- to post-treatment on symptoms of depression and generalized anxiety. Uptake of ICBT by the clinic therapists was encouraging, with continued use of ICBT within the clinics post-trial and growth in use of ICBT over time (i.e., 110 patients were treated by community therapists in first 12 months the ICBT program was implemented; 205 patients were treated by community therapists over the last 12 months).

While there is growing literature on attitudes towards ICBT [8, 9] and the effectiveness of ICBT in clinical practice settings [5], as well as on factors that impact the implementation of e-health generally [10], there is limited literature concerning models of disseminating ICBT and experiences with implementing ICBT. Lack of information on implementation could represent a barrier to wider scale implementation. In the present research, we first describe the facilitation model and strategies that were used [11] to support ICBT implementation in community mental health clinics in Saskatchewan. Second, we use the Consolidated Framework for Implementation Research CFIR; [12] to examine the perceived barriers and facilitators to ICBT implementation among therapists and managers within the community mental health clinics. The CFIR is a comprehensive meta-theoretical framework (based on a review of 19 other theories) that outlines diverse constructs that have the potential to impact program implementation. More specifically, this study explores the extent to which ICBT implementation was impacted by intervention characteristics (e.g., evidence, advantages, complexity, quality, cost), the outer setting (e.g., patient needs, networks, policies), the inner setting (e.g., structure, communication, culture), individual characteristics (e.g., therapist/manager knowledge, self-efficacy, interest), and implementation processes (e.g., planning, engaging, conducting, reflecting, and evaluating). The findings may assist others who are interested in incorporating ICBT into service delivery within community mental health clinics in that we highlight a process for ICBT implementation. We also identify facilitators that are associated with the uptake of ICBT as well as barriers that need to be overcome to improve implementation efforts. The results also contribute to growing literature on CFIR and how it can be used to understand and evaluate program implementation efforts [13].

## Methods

### Design/aim

This process evaluation aimed to identify barriers and facilitators that influenced ICBT implementation in seven community mental health clinics distributed across one province. A bespoke survey, designed around CFIR constructs, consisting of open- and closed-ended questions, was administered to therapists and managers approximately 2 years after ICBT for depression and anxiety was implemented in the clinics.

### Context and setting

#### Geographical context

This study took place in Saskatchewan, Canada, which is a landlocked province located in west-Central Canada, and has an area of ~650,000 km^2^. The population estimate in 2016 was ~1.1 million with approximately half of the population residing in two large cities over 250,000, and other residents living in small cities, towns, or rural and remote areas [14]. Most residents live in the southern prairie half of the province, while the northern forested part of the province is sparsely populated. The majority of Saskatchewan residents identify as Caucasian.

#### Healthcare system

Throughout Canada, medically necessary medical office and hospital care is publicly funded and financed by the government through taxation [15]. In addition, mental health care that is delivered within publicly funded healthcare institutions (e.g., hospitals, community mental health clinics) is also covered. Nevertheless, resources allocated to mental health care is lower than the demand for these services with many patients reporting their needs are unmet or only partially met [16]. Many individuals in Canada turn to private health insurance or personally pay for mental health services [15].

The community mental health clinics involved in the current research were funded by the provincial government to provide outpatient mental health services to patients within specific regions of the province. Table 1 describes the population served by the clinics (ranging from 31, 663 to 26, 3065 residents), the length of time clinics had delivered the ICBT at the time of the survey (19–29 months), the number of patients from each health region who received ICBT (36–386), the number of therapists from each clinic who delivered ICBT (1–13), and the number of therapists and managers from each clinic who participated in the study. Table 2 describes the external facilitation model that was used to actively aid ICBT implementation within the community clinics. Specifically, we elaborate on how a specialized ICBT unit (Online Therapy Unit; OTU) at the University of Regina facilitated ICBT implementation within the clinics, such as by developing a website and policies and procedures for delivering ICBT, training therapists in ICBT, and auditing and providing clinical feedback to therapists. Of note, OTU therapists also treated patients with ICBT, taking on patients when community clinic therapists were unavailable. At the time of the study, 385 patients had been treated by community therapists and 479 had been treated by OTU therapists.

**Table 1:** Population served, months offering ICBT, ICBT utilization and participation rate by community mental health clinic

Region of Community Mental Health Clinic	~Adult population	# of months clinic used ICBT at time of survey	# patients from clinic treated with ICBT (*n* = 864)	# of patients from clinic treated by external OTU (*n* = 479)	# patients from clinic treated by clinic therapist (*n* = 385)	# of therapists from clinic who used ICBT (*n* = 40)	# of therapists from each clinic completed survey (*n* = 22)	# of managers from each clinic completed survey (*n* = 11)
Regina Qu’Appelle	218,783	27	386	215	171	13	8	3
Saskatoon	263,065	29	213	117	96	10	6	1
Five Hills	43,261	28	91	45	46	6	2	2
Sun Country	44,870	28	40	16	24	3	1	1
Cypress	34,525	26	48	34	14	2	1	1
Kelsey Trail	31,663	20	36	27	9	1	1	1
Prince Albert Parkland	58,595	19	50	25	25	5	3	2

*ICBT* internet-delivered cognitive behavior therapy for depression and anxiety, *OTU* Online Therapy Unit

**Table 2:** External facilitation model used to facilitate ICBT implementation within community mental health clinics

Online Therapy Unit (OUT) at the University of Regina facilitated ICBT in the community clinics	The OTU was founded to provide centralized assistance with implementation of ICBT in the community clinics. The OTU created a platform and website that allowed therapists to deliver ICBT; established policies and procedures for therapists to deliver ICBT; trained therapists in the provision of ICBT; provided ongoing supervision and consultation with cases; provided technical assistance; screened patients; matched patients to therapists; monitored the service and treatment outcomes; and identified and resolved barriers to implementation. Key actions of the OTU as an external facilitator of change are outlined below.
Accessed new funding	Research funding was obtained to support the operation of the OTU.
Developed resource sharing agreement	The OTU developed a partnership with Macquarie University in order to trial a previously developed ICBT Course in Saskatchewan.
Built a coalition between OTU and community mental health clinics	Partnerships were formed between OTU and seven community mental health clinics.
Educational meetings	The OTU educated and trained therapists in the provision of ICBT.
Developed tools for promoting ICBT	The OTU developed posters, letters for physicians, and an online video to inform the community about ICBT.
Consensus discussions	Tri-quarterly meetings were held between the OTU and the Directors of the community clinics. During these meetings, positive experiences with ICBT delivery were shared. Barriers to implementation were discussed, such as: 1) how to best educate patients about ICBT; 2) which patients should be included/excluded from ICBT; 3) how to assign therapists to work with ICBT patients; 4) how to provide supervision to therapists; and 5) how to manage health records related to ICBT.
Individuated pilot by community mental health clinics	Each clinic determined the number of therapists they would train in ICBT, and the number of patients who would receive ICBT.
Monthly updates	The OTU provided monthly updates to clinics on the number of patients screened, treated, and completing ICBT.
Audit and provide feedback	The OTU audited the provision of ICBT within each of the community clinics. Feedback was provided to the therapists on methods that could be employed to improve delivery of ICBT.
Email reminders	Therapists received email reminders periodically regarding important aspects of providing ICBT (e.g., reminding therapists to complete weekly check-ins with patients, to build supportive relationships with patients, to remind patients to complete the course, to assist patients in applying ICBT content to life circumstances).

*ICBT* Internet-delivered cognitive behaviour therapy

#### Online therapy process

Table 3 describes how patients were deemed eligible for ICBT, how patients were assigned to community therapists or OTU therapists, and the specifics of the ICBT program utilized. In brief, after screening, patients were assigned to the first available therapist. The ICBT program was then accessed by patients and therapists by logging onto a secure server. On this server, ICBT materials were viewed, psychological measures were administered to track patient progress through treatment, and patients and therapists communicated via secure email. The ICBT program materials were developed by the eCentreClinic (https://www.ecentreclinic.org/) at Macquarie University, Sydney, Australia and are available through a license to the Unit. The ICBT course contains five core lessons, which patients review over 8 weeks. Using text and images presented in a slideshow format, the lessons describe: 1) the cognitive behavioral model and symptoms of depression and anxiety; 2) thought monitoring and challenging; 3) de-arousal strategies and pleasant activity scheduling; 4) graduated exposure; and, 5) relapse prevention. At the end of the lesson, patients are able to download a summary of the materials. Therapists also encourage patient completion of homework assignments to facilitate skill acquisition. Furthermore, patients read case studies that serve to demonstrate the application of skills. On a weekly basis, therapists are required to check in on patients and assist them in learning and applying the skills taught in the course. Most contact is via secure email, but phone calls can also be made if this is preferred by patients or clinically indicated.

**Table 3:** Overview of ICBT Delivery Process

Screening conducted by OTU	Online screening assessed patients for basic eligibility:(1) 18 years or older; (2) residents of Saskatchewan, Canada; (3) self-reporting symptoms of depression and or anxiety; (4) able to access and comfortable using computers and the Internet; (5) reporting no past history of psychotic symptoms; (6) available to participate in ICBT for 8 weeks; and (7) willing to provide a physician as an emergency contact. Eligible patients then completed additional online questions about their background and psychological symptoms/distress. A follow-up telephone interview was conducted by the OTU to ensure patients’ consented to ICBT and were not at high risk of suicide, needing help with a different disorder (e.g., alcohol or drugs, psychosis, bipolar), or in receipt of regular therapy.
Coordination	Patients were assigned to the first available registered therapist in one of the participating community clinics. If unavailable, the patient was offered treatment by a supervised graduate student or a registered provider working in the OTU.
Website	ICBT was available through a single secure server managed by the OTU. All patients and therapists used login credentials to access the course and communicated through this server.
ICBT Program	All patients were offered an ICBT course, called the *Wellbeing Cours*e [see 7 for details]. This is a transdiagnostic cognitive behavioural intervention targeting symptoms of depression and anxiety. It comprises 5 online lessons (text-based with visual images) released over 8 weeks that provide psychoeducation and instructions about: 1) symptom identification and the cognitive behavioral model; 2) thought monitoring and challenging; 3) de-arousal strategies and pleasant activity scheduling; 4) graduated exposure; and 5) relapse prevention. Patients are assigned homework to facilitate acquisition of skills.
Therapist-assistance	Patients were able to contact therapists by secure email for 8 weeks. Therapists responded to patient emails once a week, with a message that:1) highlighted the lesson content; 2) answered questions and assisted patients in applying skills; 3) reinforced progress, completion of the lessons, and practice of skills; and 4) provided support and normalized patient challenges. Therapists had the option to phone patients or send additional messages if they deemed this would facilitate treatment. Therapists were instructed to spend ~15 to 20 min on therapist-assistance per week.
Outcome monitoring	Patients completed brief measures of depression and anxiety [see 7 for details on measures] prior to each lesson to assist therapists in systematically assessing patient progress.

*ICBT* Internet-delivered cognitive behavior therapy, OTU Online Therapy Unit

### Participants

Participants included managers and therapists who worked in seven community mental health clinics across Saskatchewan who were involved in ICBT implementation at the time this survey was conducted. Completion of the survey was high, with 11 out of 12 managers (*n* = 3 psychologists; *n* = 5 social workers; *n* = 2 nurses; *n* = 1 administrators; *n* = 2 male; *n* = 9 female) and 22 out of 23 therapists (*n* = 7 psychologists; *n* = 11 social workers; *n* = 3 nurses; *n* = 1 counselor; *n* = 2 male; *n* = 20 female) who were contacted agreeing to participate. On average, therapists who participated had provided ICBT to 11.41 (*SD* = 7.12) patients at the time they completed the survey. Of note, we did not contact 17 therapists who provided ICBT in the past, but had retired or resigned from their position at the time of the survey.

### Procedure

Research ethics board approval for the study was obtained from the University of Regina. All participants provided electronic informed consent and were offered a $10 coffee card as a token of appreciation. The secure online survey (available in Additional file 1) was administered using the Qualtrics software program. After completing background questions, participants rated their perceptions of the importance of ICBT (e.g., whether residents should have access to ICBT, whether involvement with ICBT implementation was worth their time) on a scale from 1 “*strongly disagree*” to 5 “*strongly agree”.* Participants were then asked their opinions regarding the number of patients who should receive ICBT *(*i.e.*, “many more”, “somewhat more”, “the same number”, “somewhat fewer”, or “none”).* Participants were then provided with a description of each CFIR domain (intervention characteristics, outer setting, inner setting, individual characteristics, and implementation process) and were asked to comment on both positive and negative aspects of the domain on ICBT implementation. For example, the following question was posed: “The inner clinic setting (e.g., structure, communication, culture) can influence program implementation efforts. Please comment on any positive or negative factors within your clinic setting that you feel may have influenced the implementation of ICBT within your health region.” After providing open-ended feedback, participants rated agreement with a series of 47 statements (see Table 5 for items) related to ICBT implementation on a scale from 1 (“*strongly disagree*”) to 5 (“*strongly agree*”). The statements referenced facets of each CFIR domain. Example questions included: “*It is positive that the ICBT Wellbeing Course was developed externally and the health region did not have to develop our own ICBT program*” (Intervention Characteristics); “*My health care region is aware of the high need for mental health care”* (Outer Setting); “M*y health region has an adequate number of therapists available to deliver ICBT*” (Inner Setting); *“Therapists in my health region have adequate knowledge of ICBT”* (Individual Characteristics); and, *“We spent adequate time planning how to deliver ICBT in advance in my health region”* (Implementation Processes).

### Analyses

Responses to open-ended questions were exported from Qualtrics into NVivo Version 10, a qualitative data software tool. The CFIR constructs served as a coding framework with text classified into pre-existing categories (see http://cfirguide.org/constructs.html for definitions) using deductive principles [17] to identify the presence of such categories in the dataset. Two coders read and coded data together reaching consensus on the CFIR domain and construct. Each sentence could contain multiple codes. Alternatively, concurrent sentences of the same thematic code could be conjoined into one unit. Once initial coding was complete, the coders examined quotes assigned to each construct as a whole and any quotes that were identified as inconsistent with the designated construct were re-examined to determine the most appropriate construct. A third researcher then reviewed quotes representing each construct and identified any points of disagreement that were then resolved through consensus. In addition to coding CFIR constructs, the coders classified comments as either positive (facilitator) or negative (barrier) in tone. Following all coding, NVivo’s query function was used to calculate the total frequency of each construct. The frequency score represents the total number of times the construct was present in the survey (see Table 4 for frequencies and representative quotes). At times, participants made recommendations to improve ICBT implementation. These comments were also classified according to domain to identify CFIR domains requiring improvement. Descriptive statistics were calculated to examine responses to rating scale survey questions (see Table 5). Ratings above 4 were interpreted as positive perceptions of implementation, while ratings below 3 were interpreted as areas of potential improvement.

**Table 4:** Frequency of positive and negative comments about implementation of internet-delivered cognitive behaviour therapy

CFIR Constructs	Positive Comments			Negative Comments			All	Representative Comments
	Therapists	Managers	Total	Therapists	Managers	Total	Total	
Intervention Characteristics								
Innovation	–	0	0	–	–	0		–
Evidence strength & quality	7	3	10	2	0	2		“I work from the perspective that this is an evidenced based service.” (Manager)
Relative advantage	13	5	18	1	1	2		“They are able to get all of the CBT approach and materials without having to come in for 5–6 weeks of face-to-face appointments. Most people can’t commit to that amount of time, so we often don’t get the opportunity to walk clients through an entire CBT program in the office.” (Therapist)
Adaptability	1	1	2	8	3	11		“Some staff that offer the service have noted that the format is somewhat rigid and this does not fit well with their service delivery style.” (Manager)
Trialability	–	2	2	–	–	0		–
Complexity	–	–	0	4	2	6		“Therapeutic alliance is a little more difficult to establish when the non-verbal communication is not a part of the eq. I think it also depends on the age of the client and their communication style.” (Therapist)
Design quality and packaging	12	4	16	4	0	4		“The course has excellent material that is well thought out and organized.” (Therapist)
Cost	1	–	1	1	–	1		–
*Construct Total*			*49*			*26*	75	
Outer Setting								
Needs & resources	5	2	7	3	1	4		“It’s great to have another program clients can access when as a health region there are such long wait times for clients to be seen.” (Therapist)
Cosmopolitanism	–	4	4	–	–	0		“This is a great program. I really like the current model of delivery where U of R is the leader and the region provides counsellors to support.” (Manager)
Peer pressure	–	–	0	–	–	0		–
External policy & incentives	–	–	0	–	–	0		–
*Construct Total*			*11*			*4*	15	
Inner Setting								
Structural characteristics	–	–	0	2	2	4		“Staff changing roles and positions has had an impact.” (Manager)
Networks & communications	–	–	0	–	–	0		–
Culture	2	3	5	–	1	1		“Our staff are invested in providing good services to the public” (Manager)
Implementation climate	3	4	7	18	4	22		“The majority of in-person intakes would have a higher need based on the complexity of their cases or severity of their symptoms, therefore I have a hard time justifying the time spent on ICBT/client over in-person counselling.” (Therapist)
Readiness for implementation	8	6	14	35	13	48		“I think that there is a negative view of ICBT at this time, as it is seen as another “demand” on therapists’ time without other responsibilities being modified.” (Therapist)
*Construct Total*			*26*			*75*	101	
Individual Characteristics								
Knowledge & beliefs about the innovation	8	4	12	9	5	14		“There are several therapists who have a strong interest in ICBT and this positively influences the implementation of ICBT. ”(Therapist) “But for some reason ICBT is a bit boring as a therapist to deliver for me. And it’s exhausting. I don’t really like writing out my therapy.” (Therapist)
Self-efficacy	–	–	0	–	1	1		–
Individual stage of change	–	1	1	1	3	4		“Most of us struggle with change” (Manager)
Individual identification with organization	–	–	0	–	–	0		–
Other personal attributes	1	2	3	0	2	2		“Some are more comfortable both with computers and with the written communication methods of the program.” (Manager)
*Construct Total*			*16*			*21*	37	
Implementation Processes								
Planning	2	3	5	–	–	0		“Very well planned and organized, all positive.” (Manager)
Engaging	14	8	22	11	9	20		“A lot of patients in our region were quite aware of ICBT. It seemed that they had been hearing about it from both their personal networks and from professional caregivers that they were involved with.” (Therapist) “I just think more people could be involved in providing the service.” (Therapist)
Executing	6	3	9	2	–	2		“It seemed like ICBT was implemented fairly efficiently in our health region.” (Therapist)
Reflecting & evaluating	2	3	5	1	0	1		“We communicated the patient outcomes to staff & senior leadership in very visual undisputable ways.” (Manager)
*Construct Total*			*41*			*23*	64	
*TOTAL COMMENTS*			*143*			*149*	*292*	

*CFIR* Consolidated Framework for Implementation Research, *ICBT* internet-delivered cognitive behavior therapy, *U of R* University of Regina, *CBT* cognitive behavior therapy

**Table 5:** Therapist and manager agreement that ICBT implementation was facilitated by CFIR constructs

Questions	Therapists *M (SD) *(*n* = 22)	Managers *M (SD)* (*n* = 11)	Overall *M (SD)* (*n* = 33)	
*Intervention Characteristics*	*4.36* *(0.72)*	*4.78* *(0.37)*	*4.5* *(0.65)*	
1. It is positive that the ICBT Wellbeing Course was developed externally and the health region did not have to develop our own ICBT program	4.32 (0.95)	4.91 (0.30)	4.52 (0.83)	
2. The research evidence on the ICBT Wellbeing course is strong	4.18 *(*1.01)	4.82*(*0.41)	4.39 (0.90)	
3. ICBT has a number of advantages for clients	4.36 *(*0.95)	4.82 *(*0.41)	4.52 (0.83)	
4. It has been beneficial to be able to trial the ICBT Wellbeing Course on a small scale in our health region	3.86 (1.08)	4.82(0.60)	4.18 (1.04)	
5. It is beneficial that the ICBT Wellbeing course treats both depression and anxiety	4.73 (0.55)	4.91 (0.30)	4.79 (0.49)	
6. It is beneficial that the ICBT course involves 5 lessons spread over 8–9 weeks	4.45 (0.86)	4.55 (0.69)	4.48 (0.80)	
7. The ICBT materials are of high quality	4.73 (0.55)	4.82 (0.60)	4.76 (0.56)	
8. It is beneficial that there is no additional cost to the health region to deliver the ICBT Wellbeing course	4.23 (1.48)	4.64 (0.92)	4.36 (1.32)	
*Outer Setting Characteristics*	*3.6* *(0.52)*	*4.16* *(0.40)*	*3.79* *(0.55)*	
9. My health region is aware of the high need for mental health care	4.05 (1.25)	4.36 (0.92)	4.15 (1.15)	
10. My health region benefits from partnering with the Online Therapy Unit	4.05 (1.40)	4.73 (0.14)	4.27 (1.21)	
11. There is pressure from other health regions in Saskatchewan to implement ICBT	2.91 (0.68)	2.91 (0.83)	2.91 (0.72)	
12. There is pressure from Saskatchewan Health to implement ICBT	3.41 (0.80)	4.55 (0.52)	3.79 (0.89)	
13. My health region is aware of recommendations of other groups to implement ICBT	3.59 (0.85)	4.27 (0.79)	3.82 (0.88)	
*Inner Setting Characteristics*	*3.10* *(0.47)*	*3.74* *(0.55)*	*3.32* *(0.58)*	
14. My health region has an adequate number of therapists available to deliver ICBT	3.05 (1.33)	3.00 (1.27)	3.03 (1.29)	
15. The waiting list in my health region is long	3.77 (1.34)	3.82 (1.66)	3.79 (1.43)	
16. We had an adequate number of formal meetings to discuss ICBT within my clinic setting	2.55 (1.30)	3.91 (1.14)	3.00 (1.39)	
17. We had an adequate number of informal meetings/discussion to discuss ICBT within my clinic setting	2.77 (1.23)	3.73 (1.27)	3.09 (1.31)	
18. We have a positive clinic culture	3.77 (1.27)	4.00 (0.63)	3.85 (1.09)	
19. There is strong interest in doing things differently in my clinic	3.55 (0.96)	4.00 (0.89)	3.70 (0.95)	
20. It is easy to incorporate ICBT in our regular clinic work flow	2.64 (1.36)	3.73 (1.62)	3.00 (1.52)	
21. There is a high priority given to ICBT in my setting	3.14 (0.99)	3.82 (1.08)	3.36 (1.06)	
22. Therapists within my clinic who offer ICBT are recognized for their important work	2.36 (1.14)	3.73 (1.62)	2.79 (1.29)	
23. We have set specific goals for ICBT in my clinic setting	2.68 (1.13)	2.64 (1.21)	2.67 (1.14)	
24. We have adequate time to reflect on how ICBT is working and address challenges in my clinic setting	2.14 (1.08)	2.82 (1.25)	2.36 (1.17)	
25. Health region managers are committed to ICBT	3.50 (1.06)	4.45(0.69)	3.82 (1.04)	
26. Therapists have been given adequate time to learn and offer ICBT	3.18 (1.37)	4.00 (1.00)	3.45 (1.30)	
27. We had adequate access to information on ICBT from the Online Therapy Unit	4.36 (0.79)	4.82(0.41)	4.52 (0.71)	
*Individual Characteristics*	*3.75* *(0.64)*	*4.42* *(0.50)*	*3.97* *(0.67)*	
28. Therapists trained in my health region have adequate knowledge about ICBT	3.86 (0.94	4.27 (1.20)	4.00 (1.03)	
29. Therapists in my health region are committed to offering ICBT	2.91 (1.11)	3.82 (1.08)	3.21 (1.17)	
30. Therapists in my health region are competent to deliver ICBT	3.95 (0.95)	4.64 (0.67)	4.18 (0.92)	
31. Therapists in my health region are committed to improving the clinic	3.86(0.89)	4.73(0.47)	4.15 (0.87)	
32. Therapists in my health region have strong computer skills	4.00 (0.69)	4.36 (0.51)	4.12 (0.65)	
33. Therapists in my health region have a strong interest in learning	3.91 (0.97)	4.73(0.47)	4.18 (0.92)	
*Implementation Process*	*3.92* *(0.58)*	*4.37* *(0.37)*	*4.07* *(0.55)*	
34. We spent adequate time planning how to deliver ICBT in advance in my health region	2.59 (1.10)	3.64 (1.21)	2.94 (1.22)	
35. Therapists in my health region received adequate training in ICBT	3.86 (1.17)	4.27 (0.91)	4.00 (1.09)	
36. My health region made sure that all staff were informed about ICBT, including those who did not actually provide ICBT	3.27 (1.16)	4.00 (0.41)	3.52 (1.06)	
37. It was helpful that the Online Therapy Unit obtained research funding to support ICBT in the province	4.05 (1.05)	4.82 (0.41)	4.30 (0.95)	
38. The Online Therapy Unit developed adequate policies and procedures for delivering ICBT	4.18 (1.00)	4.82 (0.41)	4.39 (0.90)	
39. The Online Therapy Unit website is adequate for delivery of ICBT	4.45 (0.80)	4.36 (1.03)	4.42 (0.87)	
40. The advertising materials developed by the Online Therapy Unit were adequate	4.09 (0.87)	4.09 (1.04)	4.09 (0.91)	
41. The Online Therapy Unit did an adequate job screening clients for ICBT	3.95 (1.00)	4.00 (0.89)	3.97 (0.95)	
42. The Online Therapy Unit did an adequate job matching therapists and clients	3.77 (0.92)	4.18 (0.75)	3.91 (0.88)	
43. The Online Therapy Unit did an adequate job providing technical assistance	4.45 (0.74)	4.36 (0.81)	4.42 (0.75)	
44. The Online Therapy Unit did an adequate job providing clinical assistance to therapists when needed	4.36 (0.85)	4.27 (0.91)	4.33 (0.85)	
45. The Online Therapy Unit did an adequate job of treating additional clients from our health region	3.68 (0.95)	4.82 (0.41)	4.06 (0.97)	
46. The Online Therapy Unit did an adequate job of keeping our health region informed of client utilization of ICBT	4.05 (1.13)	4.82 (0.41)	4.30 (1.02)	
47. The Online Therapy Unit did an adequate job of keeping our health region informed of ICBT client outcomes	4.09 (1.02)	4.73 (0.47)	4.30 (0.92)	

*CFIR* Consolidated Framework for Implementation Research, *ICBT* internet-delivered cognitive behaviour therapy

## Results

### Perceptions of ICBT

There was strong indication that both therapists and managers had positive perceptions of ICBT. Specifically, both agreed strongly that Saskatchewan residents should have *access to ICBT* (*M* = 4.61/5, *SD* = .50), that *health regions should be committed to ensuring access to ICBT* (*M* = 4.12/5; *SD* = 1.05), that *health regions should identify barriers and facilitators associated with ICBT* (*M* = 4.12/5; *SD* = 1.05), that *health regions should continuously monitor and evaluate ICBT* (*M* = 4.06; *SD* = 1.25) and, that it was *worth their time to be involved in ICBT* (*M* = 4.39; *SD* = .79). Also reflecting positive perceptions of ICBT, ~45% of participants indicated that a *somewhat greater* number of patients should receive ICBT in the future, ~21% supported treating *about the same* number of patients, and promisingly ~15% reported that *many more* patients should receive ICBT. Very few participants indicated that the health regions were *treating too many* patients (9.1%) or *should not provide ICBT* (9.1%).

### Perceptions of implementation

Table 4 lists the frequency of positive and negative comments along with representative quotes made by participants related to each CFIR construct. Table 5 displays mean ratings on statements related to the CFIR constructs. The findings are summarized below by CFIR domain with attention given to recommendations for improving implementation as well as differences between therapists and managers.

#### Intervention characteristics

##### Qualitative findings

Overall, 26% (75/292) of participant responses concerned the impact of the intervention on implementation; the majority of these comments were positive (65%; 49/75). Both therapists and managers emphasized *the relative advantages of ICBT* over face-to-face care, noting it overcomes multiple barriers to face-to-face therapy related to time and location, and is a more convenient method of receiving care. Many participants also highlighted that the *design and packaging* of the ICBT program was excellent (e.g., brief, organized, clear, practical, user-friendly) and recognized the program as *evidence-based.*

The most common barrier related to the intervention concerned the lack of *adaptability* of the ICBT program for certain patients. Therapists reported that the ICBT program is highly standardized and not designed for blending with face-to-face care or adapted for patients who need more or less support or time to complete the program. Many participants shared it would be helpful to offer ICBT for other conditions, such as addiction and trauma. As stated by one participant: “This [ICBT] needs to continue, we need to explore other areas where we could expand this type of service broader” (Manager).

##### Quantitative findings

The mean rating for questions assessing intervention characteristics was above 4.5, also suggesting that participants viewed intervention characteristics as facilitating implementation. As with qualitative feedback, participants rated the ICBT program as having strong research evidence, multiple advantages, and being well designed and packaged. Ratings also revealed that participants felt it was beneficial that the ICBT program was externally developed, could be used to treat both depression and anxiety in a short period of time, did not result in additional costs to the health region, and could be implemented on a small scale.

#### Outer setting

##### Qualitative findings

Relative to other CFIR constructs, participants made fewer comments about how the outer setting impacted ICBT implementation (~5%; 15/292); 73% (11/15) of the comments made were positive, with many participants reporting that ICBT *aligned with patient needs for resources,* and thus facilitated implementation. Participants highlighted that is helpful to have another treatment option for patients, especially when patients are waiting for face-to-face services, or when patients are reluctant to seek in-person services related to stigma or inconvenience. There was recognition among some participants that ICBT would not meet the needs of all patients who have a strong preference for face-to-face care.

##### Quantitative findings

Ratings were consistent with qualitative comments and suggested that the outer setting had somewhat aided implementation. Ratings indicated that participants were aware of the high need for mental health care, and health regions benefit from partnering with the OTU. Other health regions were not regarded as exerting pressure to implement ICBT.

#### Inner setting

##### Qualitative findings

When queried about experiences implementing ICBT, participants most frequently described the impact of the *inner setting* on implementation (35% of all comments; 101/292). The majority of these comments (~74%; 75/101) were negative in tone suggesting the inner setting likely hindered ICBT implementation. Participants described low r*eadiness for implementation* due to *lack of available resources*. Some therapists discussed feeling pressured to deliver ICBT and that ICBT added to their current high caseloads resulting in an unmanageable workload. Several indicated that they would often deliver ICBT during lunch hours or after work. Managers echoed therapists’ sentiments, citing staff shortages and therapists’ high existing workloads as barriers to implementing ICBT. *Implementation climate* also was raised as a barrier to implementation with therapists often discussing the lower *relative priority* of ICBT compared to face-to-face therapy. As described by one manager, there is a culture that perceives face-to-face counseling as the optimal service.

While the inner setting was described primarily as a barrier to ICBT implementation, some participants reported that *leadership engagement* with ICBT facilitated implementation (e.g., “A positive factor is that there is support from management to implement ICBT” Therapist). It was also noted that having *access to knowledge* about ICBT through the OTU positively impacted implementation, such as attending a one-day workshop on ICBT prior to delivering ICBT and also being able to consult with the Unit on an ongoing basis to address any challenges in delivering ICBT. Additionally, some participants reported implementation was facilitated by the *compatibility* of ICBT with the clinic need to manage waiting lists and provide stepped care.

Some participants offered recommendations for improving the inner setting to facilitate ICBT. Numerous participants indicated that *resources* for ICBT need to be addressed, such as ensuring adequate time for ICBT (e.g., reducing face-to-face therapy patients), creating positions that predominantly deliver ICBT or, conversely, training more therapists in ICBT to reduce the ICBT workload. Several participants indicated increasing the ICBT patient load (while reducing face-to-face care) would improve therapist self-efficacy and thus ICBT effectiveness and efficiency. As another method of improving the inner setting, some participants recommended therapists be rewarded for providing ICBT (e.g., educational opportunities, monetary reward).

##### Quantitative findings

On rating scales, the average rating on inner setting items was 3.32, indicating primarily neutral impressions of the impact of the inner setting on ICBT implementation. Participants gave lower ratings to items regarding: having adequate time to reflect on ICBT and address challenges, setting specific goals and priority for ICBT, therapists being recognized for their work, ease of incorporating ICBT into the clinic workflow, adequacy of number of therapists to deliver ICBT, and adequacy of number of meetings and time to learn and discuss ICBT. Reflecting a positive tone, similar to qualitative comments, participants agreed that having adequate access to information on ICBT benefitted implementation.

#### Individual characteristics

##### Qualitative findings

Participants made relatively few comments about the impact of individual characteristics on implementation (13% of comments; 37/292); the comments were both negative (56%; 21/37) and positive (44%; 16/37). Comments reflected the perception that therapists, who had *positive beliefs* about ICBT, facilitated implementation, while therapists who held unenthusiastic beliefs about ICBT (e.g., viewing ICBT delivery as “boring” and “unrewarding”) hindered program uptake. Managers shared that staff who reported uncertainty regarding ICBT could have inadvertently transferred their uncertainty to their patients.

##### Quantitative findings

In terms of ratings, participants generally agreed that therapists had adequate knowledge about ICBT, were competent in ICBT, were committed to improving their clinics, have strong computer skills, and have a strong interest in learning. Less certainty was expressed regarding therapists’ commitment to offering ICBT.

#### Implementation processes

##### Qualitative findings

Approximately, 22% (64/292) of comments made by participants concerned implementation processes; the majority of these comments (64%; 41/64) were positive, especially in terms of *planning*, *executing,* and *reflecting and evaluating.* However, *engagement* was perceived as both facilitating and hindering implementation. For example, participants reported that ICBT was facilitated by training therapists to deliver ICBT and by educating patients and referring-providers about ICBT. Furthermore, a number of participants mentioned that “champions” who were keen to deliver ICBT facilitated implementation. Other participants noted that the promotion of ICBT to patients took considerable time. Managers’ responses reflected having difficulty engaging some therapists, referring-providers (e.g., family physicians, psychiatrists), and the general public with ICBT.

In terms of recommendations, the majority of participants indicated that ICBT requires additional promotion to the public (i.e., potential patients) and to referring-providers. Several participants also noted the need to correct misperceptions that face-to-face services are always superior to ICBT. One participant expressed, “We need to continue to have conversations with staff about its effectiveness, ensuring staff have a good understanding of the program” (Manager). In terms of ICBT training, participants recommended creating an online training program for therapists to increase the ease of access to training, and ultimately the uptake of ICBT.

Other recommendations participants shared included adjusting the model of service delivery. Some voiced that ICBT should be offered routinely to patients who are waiting or as a first step in care before face-to-face care. Others suggested alternative models of delivery to facilitate implementation, such as ICBT being provided by one stand-alone organization: “Instead of spreading this service across all of the different health regions, I think it would be more efficient to fund/resource one centralized service line to provide ICBT province-wide” (Manager). Lastly, participants expressed an interest in receiving more comprehensive updates on patient utilization of ICBT in an aesthetically pleasing manner (i.e. graphs, text boxes, photos). They specifically expressed interest in knowing if ICBT reduced the need for future face-to-face care and resulted in cost-savings.

##### Quantitative findings

Of the 14 items assessing implementation processes, the vast majority suggested that participants agreed that the OTU had a positive impact on ICBT implementation in most regards (e.g., developing a usable website for ICBT delivery, providing technical and clinical assistance, developing polices, securing funding, reporting on patient utilization and outcomes, training therapists, screening patients for ICBT, matching patients to therapists, treating additional patients when health region therapists were not available). Lower ratings, however, were obtained on the extent to which ICBT was planned in advance and on informing all staff in the health region about ICBT.

## Discussion

There is growing research demonstrating ICBT is effective for treating depression and anxiety and overcomes numerous barriers to accessing treatment [18]. Nevertheless, use of ICBT in clinical practice settings remains limited. This study fills a considerable gap in the literature by describing an external facilitation model that was used to implement ICBT within dispersed community mental health clinics in one Canadian province. We also report on contextual facilitators and barriers to ICBT implementation in order to understand factors that contributed to ICBT implementation and those that could impact future adoption and use of ICBT. Detailing our implementation strategies, as well as barriers and facilitators, may assist managers and therapists in other clinical settings who aspire to implement ICBT using a similar model.

### Facilitators and barriers

*Intervention characteristics* and *implementation processes* appeared to have the greatest positive impact on ICBT implementation. In terms of the intervention, the *relative advantages, design quality, and strength of evidence* of the ICBT program appeared to be important factors facilitating ICBT implementation. Examination of participants’ responses to rating scales revealed other aspects of the intervention that also likely facilitated ICBT implementation including the transdiagnostic nature of the program, the brevity and effectiveness of the program, and low cost and external development of the program. Processes used to implement ICBT also served to facilitate ICBT implementation, especially in terms of *engaging diverse stakeholders* in ICBT implementation (e.g., patients, therapists). Participants also reported a positive perception of the diverse actions taken by the external facilitation Unit to implement ICBT in the clinics (e.g., managing the website, providing education and technical and clinical assistance, securing external funding). In comparison, the outer setting, inner setting, and individual characteristics were less consistently regarded as having facilitated ICBT implementation.

The *inner setting* emerged as representing the greatest barrier to ICBT implementation especially in terms of lack of *readiness for implementation* and *implementation climate*. Specifically, ICBT implementation was considered to be negatively impacted by *limited resources* being allocated to ICBT and perceptions that ICBT was of *lower relative priority* compared to face-to-face therapy. Of note, while therapists, on average, treated only 11 patients, this was still deemed to be taxing and difficult to integrate with existing patient workloads. Participants also felt inadequate attention was given to *setting goals, reflecting and addressing challenges, and providing incentives and rewards* to therapists; these factors were considered to hinder implementation.

While the inner setting clearly emerged as the greatest barrier to ICBT implementation, analyses suggested that there were some additional barriers to implementation. Low *adaptability* of ICBT in terms of support and length of treatment was perceived as a barrier to implementation. Participants also indicated that ICBT implementation would have been greater if additional clinical concerns were addressed with ICBT (e.g., alcohol or drug problems). Other barriers to implementation included n*egative beliefs about ICBT* among some therapists and the need for even *greater engagement of stakeholders* in ICBT.

The results of the current study show consistency with a recent systematic review on factors that influence implementation of e-health interventions [10]. Consistent with this recent review, we found that ICBT implementation amongst mental health clinics was impacted by diverse constructs and no single construct explained implementation. Furthermore, similar to this review, we identified that the *inner setting implementation climate* negatively impacted ICBT implementation and *implementation processes, such as planning, engaging, reflecting and evaluating,* positively impacted implementation. Unique from this review, in the current study, we found that ICBT implementation was significantly facilitated by the *relative advantages of ICBT*, the *quality of the ICBT program,* and *evidence supporting ICBT*. These variables did not emerge as prominently in the review of e-health implementation; this could reflect that Ross et al. examined many forms of e-health (e.g., electronic medical records, decision support) and that intervention characteristics play a more prominent role when it comes to implementation of therapeutic programs. *Evidence supporting* ICBT may have been a particularly strong facilitator in our study as the implementation approach involved an initial workshop that incorporated evidence supporting ICBT. Furthermore, on a regular basis, we provided clinics with information on outcomes of ICBT and also regularly posted results of studies on ICBT on the Unit website. A further discrepancy is that in the past review, external policies and incentives were identified as highly beneficial for e-health implementation. This variable did not emerge in the current study, but could reflect that participants in our study were therapists and managers as compared to those involved in overseeing health policy. Of interest, in the current study *relative priority* of e-health within the inner setting emerged as a prominent barrier to implementation; Ross et al., [10] identified no past research on this construct suggesting the present study makes a unique contribution to the e-health implementation research.

Overall, we found CFIR to be a useful framework for guiding and examining multiple contextual factors impacting ICBT implementation. Similar to others, the CFIR terminology and language were coherent [12]. We did not find any need to add categories to the CFIR framework. In the future, it would be valuable to explore whether the variables that were identified as impacting ICBT implementation in the current context also explain implementation in other clinical settings. In this study, we used a bespoke survey that may prove helpful to others who are interested in evaluating implementation with respect to CFIR. This format had the advantage of being cost-effective for capturing information across multiple sites.

### Study strengths and limitations

It is critical to study ICBT from an implementation perspective given that barriers and facilitators to implementation can impact the utility and sustainability of ICBT. Most past research on ICBT has focussed on ICBT within specialized clinics; however, it is important to consider implementation within settings where it may be more challenging to offer ICBT given multiple demands on therapist time. This is the first multi-site evaluation of the factors impacting uptake of ICBT in community mental health clinics. Lessons learned from implementation studies assist with local implementation, but also support widespread adoption of an intervention, allowing others to consider the strengths and challenges of using the facilitation model to implement ICBT, and to anticipate specific barriers and facilitators to ICBT implementation [12].

An additional strength of this study is that we utilized the CFIR to understand implementation barriers and facilitators; this is a comprehensive model built on the review of many other implementation theories [12]. We used both open-ended questions and rating scales to explore implementation processes and information was collected from two perspectives (managers and therapists). While there was convergence between methods of data collection, the ratings scales appeared most useful for identifying facilitators of ICBT implementation, while the open-ended responses were helpful for understanding barriers and potential areas of improvement. A final strength is the high participation rate.

In terms of limitations, it should be noted that qualitative analyses have the potential to be subjective and influenced by coder bias. In order to offset this bias, however, multiple coders were used and quantitative ratings supplemented the qualitative findings. A further limitation of this study is the sample size which could limit generalizability of the findings. The sample size also limited our ability to examine differences in implementation experiences between managers and therapists and uncover differences between clinics with higher and lower ICBT implementation. Also of note, there was a high degree of staff turnover and 17 therapists who provided ICBT had either resigned or retired prior to conducting the survey and therefore did not participate. It should be noted that facilitators and barriers to ICBT implementation are likely linked to the model of implementation, the local population, and the health care system. For example, with different implementation models, there may be less emphasis placed on sharing evidence regarding the intervention and this factor may impact implementation in a different fashion than what was found in our study. As another example, in a different region, funded by private insurance or with better access to mental health care, different facilitators and barriers to ICBT implementation may emerge. Future research is needed to examine ICBT implementation in different settings, with different populations, using different models. The open-ended questions and rating scales were developed specifically for this study. It is possible that findings may have varied if we used a different framework [19], formulated different questions, or utilized a different method of study (e.g., focus groups). Given our interest in examining facilitators and barriers to ICBT implementation, all participants in this study were directly involved in ICBT implementation. Perceptions of ICBT were highly favorable among this group and could be quite different among providers without experience with ICBT. It should also be noted that we assessed barriers and facilitators retrospectively after participants had experience with ICBT. Given the retrospective observational methodology, it is difficult to fully disentangle which facilitators and barriers were most important.

### Future directions

There are a number of strategies that could potentially improve ICBT implementation in the future that are important for other groups to consider who have an interest in using a similar facilitation model to implement ICBT. In terms of the intervention, it would be beneficial to explore if implementation could be improved if the ICBT program was adapted to meet the needs of the patients, especially in terms of program length and amount of therapist support. Rather than offering standardized ICBT it may be valuable to explore if reach, outcomes, and implementation of ICBT could be improved if ICBT were personalized to the patient. This would be consistent with the notion of “patient-centered care”, which refers to “providing care that is respectful of and responsive to individual patient preferences, needs, and values” , p. 6 [20]. There is considerable research demonstrating that patient-centered care is associated with greater patient satisfaction and self-management [21]. Although it seems intuitive that personalized ICBT would improve adherence and outcomes, it is important to examine this systematically. Another possibility is that personalization may contribute to non-therapeutic discussion and drift away from the key tasks of ICBT, ultimately resulting in a reduction in patient adherence and outcomes.

In terms of the inner setting, it would be valuable to systematically examine if uptake could be improved if adjustments were made to resources dedicated to ICBT. Some participants suggested that ICBT should only be offered by a centralized unit while others suggested that settings other than mental health clinics should deliver ICBT (e.g., primary health care clinics). Others felt that within the clinics it would be desirable to have either fewer therapists or alternatively more therapists deliver ICBT. Still others felt that therapists should be given more time to offer ICBT. Of interest, within the OTU, consistent with how ICBT is described in the literature [18], most therapists need 15 to 20 min, per patient, per week to provide support. This reflects that ICBT materials reduce the need for therapists to provide information to patients. A number of therapists in the clinics, however, reported taking 45 min or more to provide support. This could reflect lack of experience or that therapists are having difficulties with the model of care and attempting to provide the same level of support that they provide in face-to-face care. It is possible that spending additional time on addressing inner setting barriers to ICBT implementation, such as workflow, could have resulted in greater use of ICBT beyond current levels. Past research has shown, for example, that compatibility of e-health programs with workflow positively facilitates e-health implementation [10]. In general, in the current trial, no specific goals were set for implementation, internal champions were not systematically identified, and incentives for delivering ICBT were not used; these approaches could represent additional strategies that could be examined to increase uptake in the future. It is also quite possible that addressing beliefs about ICBT, such as beliefs that face-to-face care is of greater priority than ICBT, could also serve to improve ICBT uptake in the future.

Another suggestion for improving ICBT implementation would be to explore if greater time and resources for engagement would improve uptake and use of ICBT. Among participants, there was a call to systematically examine ICBT as a first step in care prior to being offered face-to-face services. Currently, ~15% of the patients who receive ICBT report they are on waiting list for face-to-face services. It would be valuable to systematically examine whether need for face-to-face care is reduced if patients receive ICBT while waiting for face-to-face care. A previous study did not find offering ICBT to patients who were waiting for face-to-face treatment reduced the need for subsequent face-to-face care [22]. This finding requires replication, however, as the research was observational in nature. In terms of other directions, many participants, in the current study, requested more information about ICBT utilization and outcomes presented in a more engaging format. It would be valuable to examine whether providing this information would improve uptake and use of ICBT beyond current levels. Lastly, it would be valuable to examine the generalizability of the current findings to other regions in Canada or across the globe.

## Conclusions

The study contributes to understanding factors that influenced ICBT implementation within community mental health clinics and serves to identify potential strategies for improving ICBT implementation in the future. The paper also presents a survey approach, including open-ended questions and rating scales, for capturing contextual factors that underlie implementation of programs. The paper adds to growing information available on CFIR.

## Additional file

Additional file 1: Open-ended and closed-ended survey assessing CFIR constructs. (PDF 114 kb)

## Abbreviations

CFIR: Consolidated Framework for Implementation Research
ICBT: Internet-delivered cognitive behavior therapy
OTU: Online Therapy Unit
