# Supplementary material for: Implementation of internet-delivered cognitive behavior therapy within community mental health clinics: a process evaluation using the consolidated framework for implementation research
Source: BMC Psychiatry. 2017 Sep 12;17:331. doi: 10.1186/s12888-017-1496-7 (PMC5596488; doi:10.1186/s12888-017-1496-7)
Supplement: Additional file 1: — Open-ended and closed-ended survey assessing CFIR constructs. (PDF 114 kb) [file 12888_2017_1496_MOESM1_ESM.pdf]

Additional file 1: Evaluating Experiences with Internet-delivered Cognitive Behaviour Therapy (I-CBT)

In the survey that follows, you will first be asked background questions and then both closed-ended and open-ended questions about your perceptions of the implementation of Internet-delivered cognitive behaviour therapy in Saskatchewan. Please answer the following background questions.

1. Which organization do you work with?
- ❍ RQHR
- ❍ Five Hills
- ❍ Sun Country
- ❍ Cypress
- ❍ Saskatoon
- ❍ Kelsey Trail
- ❍ PA Parkland
- ❍ Saskatchewan Health

2. What role(s) do you hold in this organization? (check all that apply)
- ❑ Service Provider
- ❑ Director
- ❑ Manager
- ❑ Professional Leader
- ❑ Other (please specify) ____________________

3. What discipline do you belong to?
- ❍ Psychology
- ❍ Social Work
- ❍ Nursing
- ❍ Other (please specify) ____________________

4. Please indicate what year you obtained your highest degree:

What is your sex?
- ❍ Male
- ❍ Female

5. On a scale from 1 to 10, with 1 being nearly impossible and 10 being very easy, in your opinion, how easy do you feel it has been for therapists to deliver ICBT within your health region?

- 1
- 2
- 3
- 4
- 5
- 6
- 7
- 8
- 9
- 10

6. Please describe what you personally did to facilitate ICBT implementation within your health region.

7. In your opinion, what steps, if any, do you feel should be taken by your health region to improve patient access to ICBT in Saskatchewan?

8. Please indicate your degree of agreement with the following statements:

	Strongly Disagree (1)	Disagree (2)	Neither Agree or Disagree (3)	Agree (4)	Strongly Agree (5)
I believe it makes sense for residents of Saskatchewan to have access to ICBT as a form of care	❍	❍	❍	❍	❍
I believe it is worth my time to be involved with the implementation of ICBT in Saskatchewan	❍	❍	❍	❍	❍
I believe health regions should be committed to ensuring residents of Saskatchewan have access to ICBT as a form of care	❍	❍	❍	❍	❍
I believe health regions should take action to ensure ICBT is available to residents of Saskatchewan, identifying and overcoming barriers to delivering ICBT	❍	❍	❍	❍	❍
I believe health regions should continuously monitor and evaluate ICBT to ensure residents of Saskatchewan have access to ICBT	❍	❍	❍	❍	❍

9. Please select the statement below that best represents your opinion about the number of clients who should be treated with ICBT by therapists in your health region?

❍ In my opinion, therapists within my health region should be providing ICBT to many more clients than we have treated to date
❍ In my opinion, therapists in my health region should be providing ICBT to a somewhat greater number of clients than we have been treating to date
❍ In my opinion, therapists in my health region should continue to provide ICBT to about the same number of clients we have been treating
❍ In my opinion, therapists in my health region, have been treating too many clients with ICBT
❍ In my opinion, therapists in my health region should not be providing ICBT to clients

In your opinion, who do you think should be responsible for the delivery of ICBT in Saskatchewan?

❍ University of Regina Online Therapy Unit AND therapists from health regions who have an interest in delivering ICBT
❍ University of Regina Online Therapy Unit only
❍ University of Regina Online Therapy Unit and therapists from a small number of health regions
❍ Other, please describe ____________________

The following section will ask for your feedback about a number of factors that may have impacted ICBT implementation within your health region.

I. INTERVENTION CHARACTERISTICS: Research suggests that efforts to implement new programs can be influenced by the characteristics of the intervention that is being delivered (e.g., evidence, advantages, complexity, quality, cost). Since 2012, therapists in Saskatchewan have been trained in the Wellbeing Course, which is a 5 lesson 8-9 week Internet-delivered cognitive behaviour therapy course appropriate for both depression and anxiety. Please provide us with your feedback on this intervention. In your opinion, please describe both positive and negative aspects of the Wellbeing Course that may have impacted implementation of ICBT within your health region.

II. OUTER SETTING: Research also suggests that efforts to implement new programs can be influenced by external/contextual factors (e.g., patient needs, networks, policies). Please describe any factors outside of your organization that you believe have had a positive and or negative influence on the implementation of ICBT within your health region.

III. INNER SETTING: The inner clinic setting (e.g., structure, communication, culture) can also influence program implementation efforts. Please comment on any positive or negative factors within your clinic setting that you feel may have influenced the implementation of ICBT within your health region.

IV. CHARACTERISTICS OF INDIVIDUALS: Program implementation can be influenced by characteristics of the individuals involved (e.g., therapist/manager knowledge, self-efficacy, interest). Please comment on any individual characteristics that you believe may have positively or negatively influenced the implementation of ICBT within your health region.

V. IMPLEMENTATION PROCESSES: Program implementation can also be impacted by implementation processes (e.g., planning, engaging, executing, reflecting and evaluating). Please comment on your perceptions of positive or negative aspects of how ICBT was implemented within your health region.

I. INTERVENTION CHARACTERISTICS:

Please indicate your agreement with the following statements:

	Strongly disagree (1)	Somewhat disagree (2)	Neither agree nor disagree (3)	Somewhat agree (4)	Strongly agree (5)
It is positive that the ICBT Wellbeing Course was developed externally and the health region did not have to develop our own ICBT program.	❍	❍	❍	❍	❍
The research evidence on the ICBT Wellbeing course is strong	❍	❍	❍	❍	❍
ICBT has a number of advantages for clients.	❍	❍	❍	❍	❍
It has been beneficial to be able to trial the ICBT Wellbeing Course on a small scale in our health region.	❍	❍	❍	❍	❍
It is beneficial that the ICBT Wellbeing course treats both depression and anxiety.	❍	❍	❍	❍	❍
It is beneficial that the ICBT course involves 5 lessons spread over 8-9 weeks.	❍	❍	❍	❍	❍
The ICBT materials are of high quality.	❍	❍	❍	❍	❍
It is beneficial that there is no additional cost to the health region to deliver the ICBT Wellbeing course.	❍	❍	❍	❍	❍

II. OUTER SETTING

Please indicate your agreement with the following statements:

	Strongly disagree (1)	Somewhat disagree (2)	Neither agree nor disagree (3)	Somewhat agree (4)	Strongly agree (5)
My health region is aware of the high need for mental health care	❍	❍	❍	❍	❍
My health region benefits from partnering with the Online Therapy Unit at the University of Regina	❍	❍	❍	❍	❍
There is pressure from other health regions in Saskatchewan to implement ICBT	❍	❍	❍	❍	❍
There is pressure from Saskatchewan Health to implement ICBT	❍	❍	❍	❍	❍
My health region is aware of recommendations that have been made by some groups to implement ICBT	❍	❍	❍	❍	❍

III. INNER SETTING:

Please indicate your agreement with the following statements:

	Strongly disagree (1)	Somewhat disagree (2)	Neither agree nor disagree (3)	Somewhat agree (4)	Strongly agree (5)
My health region has an adequate number of therapists available to deliver ICBT	❍	❍	❍	❍	❍
The waiting list in my health region is long	❍	❍	❍	❍	❍
We had an adequate number of formal meetings to discuss ICBT within my clinic setting	❍	❍	❍	❍	❍
We had an adequate number of informal meetings/discussions on ICBT within my clinic setting	❍	❍	❍	❍	❍
We have a positive clinic culture	❍	❍	❍	❍	❍
There is strong interest in doing things differently in my clinic	❍	❍	❍	❍	❍
It is easy to incorporate ICBT in to our regular clinic work flow	❍	❍	❍	❍	❍
There is a high priority given to ICBT in my setting	❍	❍	❍	❍	❍
Therapists within my clinic who offer ICBT are recognized for their important work	❍	❍	❍	❍	❍
We have set specific goals for ICBT in my clinic setting	❍	❍	❍	❍	❍
We have adequate time to reflect on how ICBT is working and address challenges in my clinic setting	❍	❍	❍	❍	❍
Health region managers are committed to ICBT	❍	❍	❍	❍	❍
Therapists have been given adequate time to learn and offer ICBT	❍	❍	❍	❍	❍
We had adequate access to information on ICBT from the Online Therapy Unit	❍	❍	❍	❍	❍

IV. CHARACTERISTICS OF INDIVIDUALS:

Please indicate your agreement with the following statements:

	Strongly disagree (1)	Somewhat disagree (2)	Neither agree nor disagree (3)	Somewhat agree (4)	Strongly agree (5)
Therapists trained in my health region have adequate knowledge about ICBT	❍	❍	❍	❍	❍
Therapists in my health region are committed to offering ICBT	❍	❍	❍	❍	❍
Therapists in my health region are competent to deliver ICBT	❍	❍	❍	❍	❍
Therapists in my health region are committed to improving the clinic	❍	❍	❍	❍	❍
Therapist in my health region have strong computer skills	❍	❍	❍	❍	❍
Therapists in my health region have a strong interest in learning	❍	❍	❍	❍	❍

V. IMPLEMENTATION PROCESSES:

.Please indicate your agreement with the following statements:

	Strongly disagree (1)	Somewhat disagree (2)	Neither agree nor disagree (3)	Somewhat agree (4)	Strongly agree (5)
We spent adequate time planning how to deliver ICBT in advance in my health region	❍	❍	❍	❍	❍
Therapists in my health region received adequate training in ICBT	❍	❍	❍	❍	❍
My health region made sure that all health region staff were informed about ICBT, including those who did not actually provide ICBT	❍	❍	❍	❍	❍
It was helpful that the Online Therapy Unit obtained research funding to support ICBT in the province	❍	❍	❍	❍	❍
The Online Therapy Unit developed adequate policies and procedures for delivering ICBT	❍	❍	❍	❍	❍
The Online Therapy Unit website is adequate for delivery of ICBT	❍	❍	❍	❍	❍
The advertising materials developed by the Online Therapy Unit were adequate (e.g video, posters)	❍	❍	❍	❍	❍
The Online Therapy Unit did an adequate job screening clients for ICBT	❍	❍	❍	❍	❍
The Online Therapy Unit did an adequate job matching therapists and clients	❍	❍	❍	❍	❍
The Online Therapy Unit did an adequate job providing technical assistance	❍	❍	❍	❍	❍
The Online Therapy Unit did an adequate job providing clinical assistance to therapists when needed	❍	❍	❍	❍	❍
The Online Therapy Unit did an adequate job of treating additional clients from our health region	❍	❍	❍	❍	❍
The Online Therapy Unit did an adequate job of keeping our health region informed of client utilization of ICBT	❍	❍	❍	❍	❍
The Online Therapy Unit did an adequate job of keeping our health region informed of ICBT client outcomes	❍	❍	❍	❍	❍

Do you have any additional feedback related to the implementation of ICBT in Saskatchewan?
